# The Complementary Health Approaches for Pain Survey (CHAPS): Validity testing and characteristics of a rural population with pain

**DOI:** 10.1371/journal.pone.0196390

**Published:** 2018-05-02

**Authors:** Termeh Feinberg, Dina L. Jones, Christa Lilly, Amna Umer, Kim Innes

**Affiliations:** 1 Department of Family and Community Medicine, Center for Integrative Medicine, University of Maryland School of Medicine, Baltimore, Maryland, United States of America; 2 Department of Epidemiology, West Virginia University School of Public Health, Morgantown, West Virginia, United States of America; 3 Department of Orthopaedics, West Virginia University School of Medicine, Morgantown, West Virginia, United States of America; 4 Division of Physical Therapy, West Virginia University School of Medicine, Morgantown, West Virginia, United States of America; 5 Injury Control Research Center, West Virginia University School of Public Health, Morgantown, West Virginia, United States of America; 6 Department of Biostatistics, West Virginia University School of Public Health, Morgantown, West Virginia, United States of America; 7 Department of Pediatrics, West Virginia University School of Medicine, Morgantown, West Virginia, United States of America; 8 Center for the Study of Complementary and Alternative Therapies, University of Virginia Health System, Charlottesville, Virginia, United States of America; Unviersity of Sheffield, UNITED KINGDOM

## Abstract

**Objectives:**

Little is known about patterns and correlates of Complementary Health Approaches (CHAs) in chronic pain populations, particularly in rural, underserved communities. This article details the development and implementation of a new survey instrument designed to address this gap, the Complementary Health Approaches for Pain Survey (CHAPS).

**Design:**

Following pilot-testing using pre-specified criteria to assess quality and comprehension in our target population, and after feedback regarding face-validity from content experts and stakeholders, the final cross-sectional self-report survey required 10–12 minutes to complete. It contained 69 demographic, lifestyle and health-related factors, and utilized a Transtheoretical Model (TTM) underpinning to assess short- and long-term use of 12 CHAs for pain management. Twenty additional items on pain severity, feelings, clinical outcomes, and activities were assessed using the Short-Form Global Pain Scale (SF-GPS); Internal reliability was assessed using Cronbach’s alpha.

**Settings/location:**

Investigators conducted consecutive sampling in four West Virginia pain management and rheumatology practices.

**Participants:**

301 Appalachian adult patients seeking conventional care for pain management.

**Results:**

Response rates were high (88% ± 4.1%). High quality and comprehension deemed the CHAPS an appropriate measurement tool in a rural population with pain. Missing data were unrelated to patient characteristics. Participants predominantly experienced chronic pain (93%), had five or more health conditions (56%, Mean = 5.4±3.1), were white (92%), female (57%), and middle-aged (Mean = 55.6 (SD = 13.6) years). Over 40% were disabled (43%) and/or obese (44%, Mean BMI = 33.4±31.5). Additionally, 44% used opioids, 31% used other prescription medications, and 66% used at least one CHA for pain, with 48% using CHAs for greater than 6 months. There was high internal reliability of the SF-GPS (alpha = .93) and satisfactory internal reliability for each of the five TTM stages across (all) twelve CHAs: precontemplation (0.89), contemplation (0.72), preparation (0.75), action (0.70), and maintenance (0.70).

**Conclusions:**

The CHAPS is the first comprehensive measurement tool to assess CHA use specifically for pain management. Ease of administration in a population with pain support further use in population- and clinic-based studies in similar populations.

## Introduction

Chronic pain, defined as pain persisting beyond normal tissue healing time of 3–6 months[1], affects over 25 million adults in the U.S. each year[2]. Unrelieved pain results in decreased physical function, longer hospital stays, and increased rates of re-hospitalization and outpatient visits[3], leading to lost employment, income, and insurance coverage. Although measurement tools exist to enable physician-patient communication regarding specific components of pain (e.g., frequency and severity)[4, 5], there is a need to assess the reliability of existing, brief measurement tools such as the Short-Form Global Pain Scale (SF-GPS), which incorporate quality of life factors often impacted by the presence of chronic pain[6].

People with some pain conditions have increased risk for comorbid pain disorders [7], which may add complexity to pain management plans. Current treatment methods for pain are largely inadequate, and non-opioid medications such as Nonsteroidal Anti-Inflammatory Drugs (NSAIDs) carry side effects for as many as 25% of long-term users[8]. In addition to the potential harm induced by NSAIDs and other non-opioids, a worrisome trend of increased prescription and utilization of opioid treatment for chronic nonmalignant pain has emerged over the past decade[9], despite accompanying physical and psychological dependence. Further, age-adjusted deaths involving opioids have more than quadrupled since 1999[10]. In particular, West Virginia’s overdose death rate has been climbing (2010 and 2014: 28.9 and 35.5 per 100,000, respectively), and was more than double the national rate, which has also been rising over time (2010 and 2014: 12.4 and 16.1 per 100,000)[11, 12]. A 2010 Pain Report from The Institute of Medicine stresses the importance of increased research regarding the translation of effective treatments for chronic pain into practice[3], and the Centers for Disease Control and Prevention recommends non-opioid pharmacologic therapy be combined with non-pharmacologic therapy to reduce pain and improve function to provide greater benefit[13]. Thus, the potential of non-pharmacological therapies for chronic pain management has garnered recent research interest[14–16].

Pain-related disorders are the most commonly reported conditions for which patients use Complementary Health Approaches (CHAs)[17–19]; these include natural products (e.g., vitamins, herbs, probiotics, etc.) and Mind-body practices (e.g., yoga, meditation, etc.), as well as Naturopathy, Traditional Chinese Medicine, and others. Although their use is considered somewhat controversial, at least 30% of the U.S. population uses CHAs[20]. Further, most people use CHAs with conventional treatments[21–23]. The most comprehensive economic review to date (N = 28 trials) concluded that substituting a CHA in place of usual care had better health outcomes and lower costs than usual care alone for a variety of chronic health conditions[24]. However, studies regarding patterns and correlates of CHA use in chronic pain populations remain relatively few, as does research regarding the overlap of CHA use and/or conventional treatments among those with pain conditions[25]. Further, little is known regarding the relation of specific demographic, lifestyle, and health-related factors to CHAs used specifically for pain; epidemiologic research is particularly sparse in Appalachian and other underserved communities. Many of these communities have the highest rates of pain conditions in the nation, including arthritis[26], yet have a rich anecdotal history of using natural products for pain management[27–30].

Numerous measurement tools have been developed to collect information regarding population-level CHA use in the U.S.[31–44]. However, only two have assessed CHAs used specifically for pain; of these resource-intensive telephone-based surveys, one lacked the identification of different CHA categories in item design[40], while another lacked temporal specification [39]. Further, no measurement tools have assessed short- and long-term concurrent use of CHAs, which is an important consideration due to the heterogeneity of potential effects for individual approaches among those with one or more pain conditions. Existing, available tools measure use of CHAs, including intake of dietary supplements alone[45–50], and those using a variety of CHAs for any purpose but not specifically for pain within 30 days[33, 34], 3 months[35], 12 months[31–37, 41–44], and at any point in an individual’s lifetime[33, 34, 36]. Though otherwise helpful, these assessments do little to provide differentiation between pain-related and non-pain use; attributing the overall use of CHAs to those with diagnosed pain conditions alone likely results in the misclassification of outcomes, resulting in biased estimates regarding the prevalence of CHAs used specifically for pain and related symptoms. In addition, participant use of CHAs for specific health conditions is captured by the largest ongoing national survey [33, 34]. However, participants may perhaps be less likely to report their CHA use for one or more specific complex pain conditions and more likely to report only for associated individual symptoms. The resulting variability to pain-specific prevalence estimates, coupled with a lack of short and self-administered tools to assess CHA use specifically for pain rather than by disease or overall use, indicates a gap in our knowledge regarding CHA use for pain. Because the utilization of CHAs for pain may be associated with potential behavior change regarding the use of opioids, a short measurement tool which utilizes a behavioral theoretical underpinning and assesses distinct stages of behavior change regarding CHAs used specifically for pain is warranted.

The Transtheoretical Model (TTM) is a behavioral theory which proposes that changes in a health behavior consist of movements between sequences of discrete, qualitatively distinct stages, characterized by distinct mindsets. Stage progression of behavior change occurs through 6 separate ‘Stages of Change,’ for which specific social-cognitive factors influence stage progression[51]: *Precontemplation* (a person has no intention to change a health behavior), *Contemplation* (they begin to consider changing a health behavior), *Preparation* (they intend to change this behavior), *Action* (initiation of the new health behavior), *Maintenance* (execution of the health behavior, sometimes for more than 6 months)[52], and the recently added *Termination* (desired health behavior complete)[53]. The ‘Stage of Change’ construct is central to the TTM[54], and has demonstrated success in cross-sectional research on diet[55, 56], the use of health care proxies[57], exercise in pain patients[58], physical activity/body satisfaction[59], and cancer prevention behaviors[60]. The Stages of Change serves as an ideal framework to consider as development guidance for the measurement of CHAs used for pain, since differing stage status may indicate varying levels of influence on conventional treatment and may also reflect different risks to patient safety. Differing stage status, which may be obtained by asking participants about health behaviors by differing lengths of time (each corresponding to a specific stage), may aid in understanding distinctions between short- and long-term use of CHAs for pain, an area which has been largely unexplored.

Information on patient CHA use is not routinely or systematically collected in the clinic setting [61, 62], and patients often do not disclose concurrent use of CHAs to their physicians[63, 64], although such CHA use may influence conventional treatments and pose risks to patient safety [65]. Thus, adverse events may be under-reported to relevant databases (i.e., the U.S. Food and Drug Administration Adverse Events Reporting System). Alternatively, the potential therapeutic benefits of short- or long-term use of certain CHAs as primary or adjunctive treatments for pain management may remain largely unrecognized. Thus, improved understanding regarding not only the patterns of CHA use in patients with chronic pain, but the risks and benefits associated both with short- and long-term CHA use and with concurrent use of specific CHAs and medications is clearly needed. An easy to use, validated instrument that captures key information on patient CHA use, which can be readily administered by health care staff in a clinical setting, would provide an effective way to address the challenges of patient non-disclosure [66–77] and aid physicians in better assessing the risks and benefits of CHA use in patients with chronic pain. A validated instrument capturing short- and long-term CHA use would improve understanding and therapeutic potential in this population, and ultimately, help reduce risk for adverse events and optimize pain management. Due in part to its demonstrated success measuring changes in various health behaviors, the TTM Stages of change serves as an ideal underlying theoretical framework to gauge the prevalence and patterns of short- and long-term CHA use for pain management, and perhaps further assess the “readiness” or receptivity of a patient with respect to non-pharmacologic therapies for pain management.

The overall aim of this study was to create and validate the first systematically-designed instrument to assess sociodemographic and health factors by CHA use for pain. This aim was achieved by accomplishing the following objectives:

1. Design a new measurement tool to capture short- and long-term use of CHAs used specifically for pain among adult patients seeking conventional care for pain management;
2. Assess face validity of this tool among content experts and stakeholders, followed by pre-testing and implementation of survey in an Appalachian population experiencing pain; and,
3. Implement and assess reliability of the SF-GPS and the CHAs by TTM stage.

## Materials and methods

### Scope of Project

**Fig 1:**
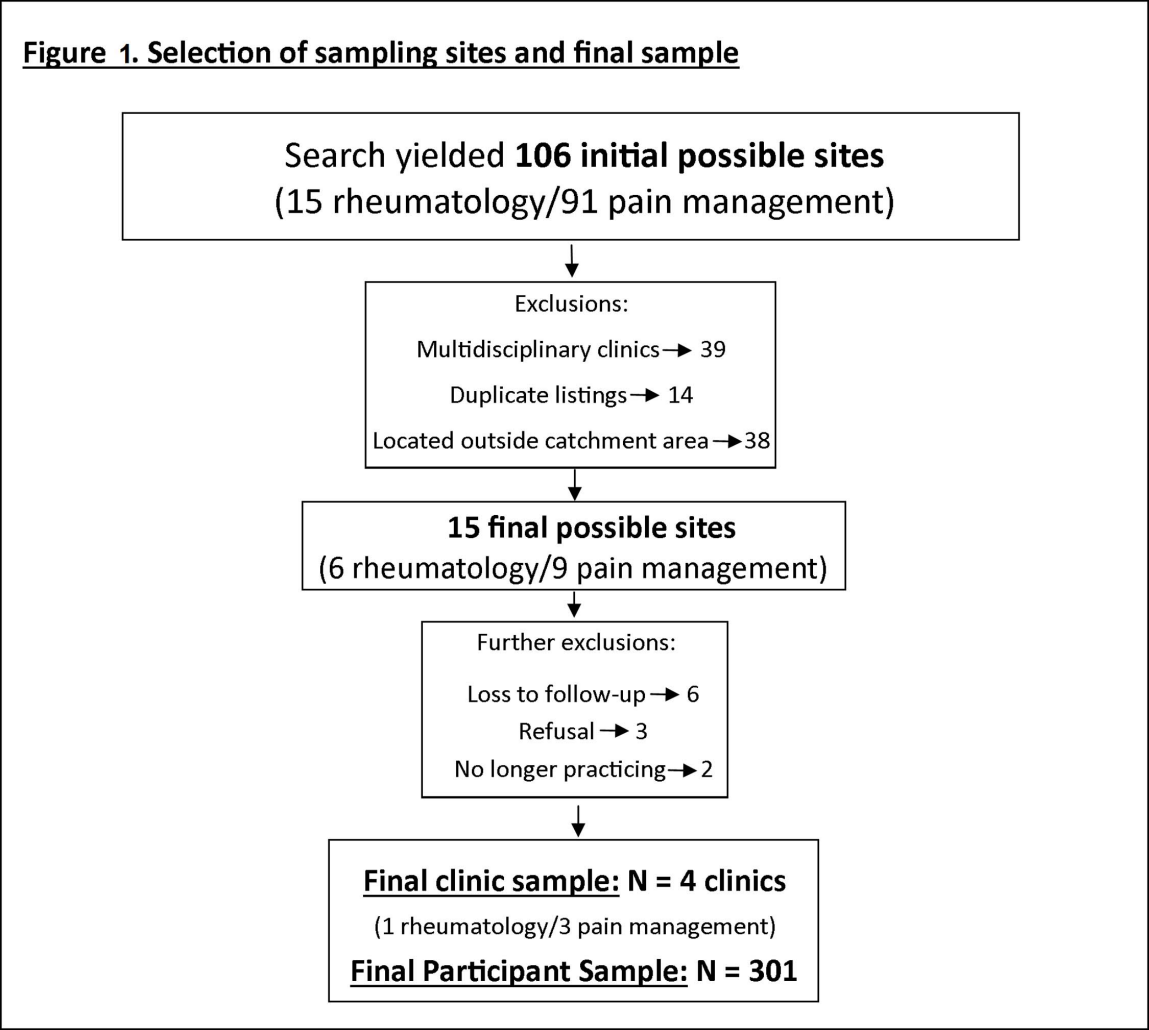
The study flow chart.

This cross-sectional survey study was conducted from June 2014 through March 2016 in a sample of 301 English-speaking adult (≥18 years) patients in four Northern WV pain and rheumatology clinics (Fig 1). We conducted primary data collection using a new measurement tool, the Complementary Health Approaches for Pain Survey (CHAPS) (S1 Appendix; CHAPS dataset and codebook are freely available as S1 Dataset and S1 Codebook), and included use of the SF-GPS[6] to measure pain severity and related characteristics. The CHAPS was implemented using consecutive sampling (response rate across 343 patients in all clinics = 88.0%) to investigate the associations between a variety of demographic, lifestyle, and health factors with 12 separate CHAs for pain management in a population seeking conventional care for pain. This study was deemed exempt by the West Virginia University IRB (#1403248198). Details regarding survey development and content, study population, and survey administration are below.

### Measurement tool

#### Underpinning: Transtheoretical Model

Patient engagement of CHA use may be considered as a health behavior [78]. Thus, we employed the Transtheoretical Model (TTM) as a guide for survey development with regard to CHA-specific questions. As far as we know, the TTM Stages of Change have not yet been applied to measure the health behavior of CHA use. Because chronic pain is defined as pain lasting from 3–6 months [1], and many CHAs likely have long-term rather than short-term (e.g., two weeks) effects [79], a 6-month cutoff was established as an appropriate collective short-term and long-term cutoff which could be used for each separate approach. Further, the 6-month cutoff has been used in previous TTM-based survey development for other health behaviors as described [55–60].

We adapted each stage to represent differing lengths of time participants used each CHA[54, 80] for pain based on previous literature[81]: “I do not know what this is;” “No, and I do not intend to within 6 months (Precontemplation);” “No, but I intend to within 6 months (Contemplation);” “No, but I intend to within 30 days (Preparation);” “Yes, and I have for less than 6 months (Action; i.e., short-term CHA use);” and, “Yes, I have for more than 6 months (Maintenance; i.e., long-term CHA use).” Additionally, researchers have indicated projects applying the TTM should include investigation of problem severity[80]; our approach to measuring problem severity of pain was through the administration of the SF-GPS[6] alongside our TTM-based CHA tool. Ideally, the CHAPS was designed to accompany a variety of pain measurement tools, since their use is not uniform in practice across disciplines.

**Survey Development.** Initial development of the CHAPS incorporated input from a panel, including: CHA researchers, survey methodologists, a clinical therapist specializing in opioid-dependence, a psychologist, and members of the WV population. The CHAPS included 2 closed-ended items regarding past and present chronic pain, with an accompanying definition as any pain lasting more than 12 weeks; demographics and lifestyle questions (10 and 6 items, respectively); previous diagnosis of 24 health conditions (including nine known to cause pain) and other health factors (3 items); 12 separate CHAs using the TTM underpinning as described, and, open-ended questions regarding specific herbs and other CHAs we may have inadvertently omitted (2 items). Our list of CHAs was derived by visually scanning the National Institutes of Health National Center for Complementary and Integrative Health (NIH NCCIH) website[82] and existing cross-sectional surveys on overall CHA use [31–44] to create a master list of relevant terms, followed by the elimination of CHAs we believed would not be accessible in some rural settings (i.e. Ayurvedic treatments, Naturopathy) or those which may be easily misinterpreted (i.e., inclusion of a commercial caloric restriction program such as Weight Watchers® as ‘special diets’). Previous psychometric testing of NHIS CHA categories in a national sample using CHAs determined that overlap of individual approaches into broader categories (i.e., Mind-body) was present[83]; when further considering our list was already limited to individual approaches for which accessibility was likely to be an issue, we concluded that we would not create broader definitions for separate group classifications. We inquired about natural products ‘Herbs/Botanicals’ (also using a definition from the NIH NCCIH), ‘Vitamins/minerals,’ *‘*Probiotics,’ and ‘Other Natural products,’ as well as ‘Acupuncture,’ ‘Massage therapy,’ ‘Spinal manipulation/Chiropractic,’ ‘Tai Chi/Qi Gong,’ ‘Yoga,’ ‘Meditation,’ ‘Other relaxation practices,’ and ‘Movement therapies.’ Accompanying examples (e.g., “Herbs…*such as Echinacea*, *Black Cohosh*, *etc*.”*)* were also included to minimize potential misclassification and ensure the incorporation of many self-administered and all core practitioner-based CAM therapies recommended for inclusion by the Members of the International Society for Complementary Medicine Research[84]. We did not incorporate specific questions about marijuana use due to sensitivity concerns. For conciseness, we did not ask about energy healing or specific religious practices, for which a wide variety of types exist. Twelve optional, nested questions regarding perceived efficacy of CHAs were also included.

**Data Collection Protocol.** CHAPS data collection protocols were standardized before implementation, and team members were given opportunities to practice engaging in dialogue before approaching patients. A cover page was attached to every survey, indicating the intent of the research study and ensuring anonymity; return of the survey served as implied informed consent. The CHAPS was designed to be a ninth grade reading level or below according to the Automated Readability Index[85], and require no more than 15 minutes to complete. Study personnel observed the timing of ≥75% administered surveys; the average time for survey completion of CHAPS and the SF-GPS among clinic patients was approximately 12 minutes. This was shorter than our anticipated response time, which may have been attributed to the recurring item format and underutilization of optional efficacy questions within the questionnaire.

**Measurement of Pain.** The SF-GPS was developed from the previously-validated Global Pain Scale (GPS) by its original authors[6] to meet the need for a simplified instrument for use by clinicians and researchers, while incorporating key elements of many existing pain assessments; these scales were created to capture the multidimensional effects of pain. The original 33-item GPS and 20-item SF-GPS[86] use a variation of the Visual Analog Scale; numbers 0–10 are spaced equidistant from one another on a horizontal line, both ends are defined as the extreme limits of the parameter (pain severity, etc.), and orientation is from the left (best) to the right (worst), indicating the impact each measure has had in a participant’s life. The SF-GPS contains 5 items each on pain (‘No Pain’ = 0 to ‘Extreme Pain’ = 10 for each: Current pain; best, worst, and average pain in past week; and, average pain in past 3 months), feelings in past week (‘Strongly Disagree’ = 0 to ‘Strongly Agree’ = 10 for each: Afraid, depressed, tired, anxious, and stressed), clinical outcomes in past week (‘Strongly Disagree’ = 0 to ‘Strongly Agree’ = 10 for each: Trouble sleeping, trouble feeling comfortable, was less independent, was unable to work, and needed to take more medication), and inability to engage in activities during past week (‘Strongly Disagree’ = 0 to ‘Strongly Agree’ = 10 for each: Go to store, do chores at home, enjoy friends and family, exercise (including walking), and participate in favorite hobbies). In a population of adults with chronic pain, the GPS demonstrated high internal reliability (alpha = .89), appropriate factor loadings within subscales (>.40), and moderate-strong correlation with similar subscales of the Short-Form McGill Pain Questionnaire (GPS Pain subscale with ‘Present Pain Intensity’ = .65), the West Haven-Yale Multidimensional Pain Inventory (GPS Pain subscale with ‘Pain Severity’ = .79 and ‘Interference’ = .54; GPS Emotions subscale with ‘Negative Mood’ = .67 and ‘Self Control’ = -.53; GPS Clinical Outcomes subscale with ‘Pain severity’ = .41), and the Perceived Stress Scale (GPS Emotions subscale = .62)[6]. The SF-GPS is freely available and has been described in greater detail elsewhere[6].

### Survey implementation

**Pilot testing period.** After obtaining provider permission and obtaining exemption status from the West Virginia University IRB, we piloted the survey in a convenience sample of pain patents attending the WVU Medicine Pain clinic in Morgantown, WV (June 2014 through March 2015). We placed a stack of surveys, pens, and a survey drop box in the waiting room; during the following 9 months, we visited every 2–3 weeks to collect surveys, and ensure surveys and pens were replenished. A total of 66 patients completed surveys during this period, including 28 during one in-person visit, where we solicited patient participation in the survey.

This preliminary version of the CHAPS was also pilot-tested in 11 patients by two investigators to assess comprehension and quality, including clarity and recall[87]. Items and survey formatting were evaluated during a 5-minute interview using nine pre-determined questions pertaining to five fundamental components of the effectiveness of a survey[88]. These included: 1) attractiveness, 2) comprehension, 3) acceptability, 4) self-involvement, and 5) persuasion (Table 1). Based on responses and other feedback, and after learning more about the patient experience through a 20-hour clinical immersion (accompanying a lead physician in patient rooms at the WVU Medicine Pain clinic), we made minor formatting changes to the CHAPS to improve clarity, and included (each) an additional question about prescription drug use, medical diagnoses we may have overlooked, perceived CHA efficacy, and military status. We then piloted this final survey version (described below) in 11 additional patients at the same clinic location.

**Table 1:** Participant feedback regarding the effectiveness of the Complementary Health Approaches for Pain Survey (CHAPS) in a subgroup of patients seeking conventional care for pain management using questions to assess negative feedback regarding attractiveness, comprehension, acceptability, self-involvement, and persuasion of measurement items (N = 22).

Questions asked by interviewers	Negative feedback
Description of chronic pain too long?^1^^,^ ^2^^,^ ^3^^,^ ^4^^,^ ^5^	<5%
Provided examples for Complementary Health Approaches understandable and easy to answer? ^1^^,^ ^2^^,^ ^3^^,^ ^4^^,^ ^5^	<5%
Clarity of efficacy question in the Complementary Health Approaches table^1^^,^ ^2^^,^ ^4^^,^ ^5^	50%; necessitated simple format change (shown in S1 Appendix)
Feedback elicited for open-ended herbal question^1^^,^ ^2^^,^ ^3^^,^ ^4^^,^ ^5^	<10%
Does “Other” option for Gender question require explanation?^1^^,^ ^2^^,^ ^3^	22%; we determined no changes to Gender item were necessary
Weighting on boxes for demographic questions^1^	No consistent preference for light vs. dark weighting
Marital status options understandable?^1^^,^ ^2^^,^ ^3^^,^ ^4^^,^ ^5^	10%; synonyms suggested
Additional options (i.e., ‘Caregiver’) included in Work status question?^1^^,^ ^2^^,^ ^3^^,^ ^4^^,^ ^5^	Nearly 60% indicated ‘Caregiver’ could be added; we determined no item changes necessary since other feedback indicated ‘Homemaker’ definition overlaps with ‘Caregiver’
Language or format of exercise question confusing? ^1^^,^ ^2^^,^ ^4^^,^ ^5^	<10%

^1^Overall attractiveness (visual appeal) assessed

^2^Comprehension assessed (Prompts included: “Do you understand what the question is asking? Are you retaining the idea of the question?”)

^3^Acceptability assessed (Prompts included: “Is question offensive or are there cultural/other barriers to answering question in this setting?”)

^4^Self-involvement assessed (Prompts included: “Would it be relatively easy to answer when thinking about your own lifestyle?”)

^5^Overall persuasion assessed (Prompts included: “Are questions relative to your concerns or conditions? Are items logically sequenced?”)

**Final Version.** Panel members who provided initial input regarding CHAPS development were re-approached to assess face validity of the final survey version. The final CHAPS survey comprised 69 self-report items and used an easily-identifiable response pattern. Commensurate to the preliminary version, items included demographic, lifestyle, and health factors (10, 6, and 25-items, respectively) and questions regarding CHAs used for pain; the latter were structured in a manner consistent with surveys utilizing a Stages of Change approach[55, 57–59, 81]. Additionally, the CHAPS incorporated 4 open-ended questions regarding additional CHA use, diagnoses, and prescriptions used for pain. The final survey version was implemented in 235 additional participants. With the addition of the 20-item SF-GPS[6], survey administration required 10–12 minutes.

#### Target study population and identification of sampling locations

Site selection is described in Fig 1. Our target study population was comprised of adult patients seeking conventional care for pain, and attending WV pain management or rheumatology clinics within a 100-mile radius of Morgantown, WV. Rheumatology clinics were selected for inclusion due to their high prevalence of patients with chronic pain[89]. Pain and rheumatology clinics were identified by conducting internet searches for physicians within 100 miles of the CHAPS office (May through July 2015). Due to rurality and limited resources, it would not have been feasible to stratify sampling of clinics from specific areas in WV.

Of the 15 and 91 rheumatology and pain management practices identified, respectively, non-pain specializing physicians were excluded, as were multidisciplinary practices including specialties other than pain or rheumatology; also excluded were opthamologists, podiatrists, neurosurgeons, chiropractors, acupuncturists, and weight centers, leaving 9 and 30 eligible rheumatology and pain clinics, respectively. After exclusion of duplicates, we engaged clinic managers or lead physicians from each clinic in an effort to describe the study, secure permission to visit, and administer the survey to patients in waiting rooms. A total of 4 eligible clinics (1 rheumatology and 3 pain management) located in 4 West Virginia counties agreed to participate. Managers from each participating clinic signed a consent form which briefly described the study purpose, stated study investigators would neither disrupt clinic practice nor provide any medical advice, and provided IRB information (#1403248198) and the primary contact information for the CHAPS study coordinator. Our resources allowed for 24 total clinic visits throughout the study.

#### Clinic visits

Our presence was not advertised in clinics. All surveys were self-administered with the exception of 14 (4.7%), which were completed by a proxy; we also assisted with personal interview format requests (n = 6, 2.0%). Upon return, each survey was stored in a locked filing cabinet in our research coordinator’s office. During all phases of data collection, we also engaged with clinic administrative staff, ensuring they were familiar with our study objectives; this facilitated return of surveys taken back into the patient rooms.

### Statistical analysis

Feedback from nine pre-determined questions relating to quality/comprehension of both survey versions was elicited, and Fisher’s chi-square tests were used to examine differences in responses across versions and clinic location. We did not conduct internal reliability or factor analysis for all the Stages of Change-based CHA items combined due to inherently distinct differences between stages[81]. However, we were able to use Cronbach’s alpha to establish internal reliability on each Stage of Change subscale, each with 12 CHA items, for whether a participant was in that particular Stage of Change (coded yes/no). Cronbach’s alpha was also used to establish the internal reliability of the total SF-GPS, in addition to each 5-item subscale Pain, Clinical outcomes, Activities, and Feelings as described above.

In order to assess if our tool was appropriately written to discourage systematic item-missing patterns, we examined the final dataset for missing data patterns (PROC MI; SAS 9.4, Cary, NC) using the missing data indicator matrix for missing data patterns[90, 91]; We did this in order to examine the most- to least-common missing data patterns by item for patterns consistent with non-response, sensitive item(s), and attrition. Consistent missing data patterns would indicate a missing data mechanism such as missing not at random (MNAR) data, which would indicate multiple imputation (MI) to be inappropriate for recovering missing information. Missing data indicators were also examined in conjunction with present demographic data, again to assist in determining presence of MNAR data. Once we determined the presence of missing at random or completely at random data instead of MNAR data, we conducted sensitivity analyses in order to determine whether there were differences in data with MI in the relationships between all sociodemographic, lifestyle, and health factors with CHA use using logistic and linear regression using PROC MI ANALYZE.

## Results

Response rates were high (88% ± 4.1%) ranging from 84 to 94% in the four sites sampled. Specific reasons for refusal included reluctance to fill out paperwork (18.8%), hand pain (12.5%), or visual impairment (12.5%).

Overall, quality/comprehension of both survey versions was strong (Table 1). The only changes which occurred between versions were simple font format changes (i.e., darker, bolder print) to ensure readability, and the addition of new questions to capture information we believed we initially overlooked (Veteran status, etc.); there were no changes to the content or wording of questions themselves. Further, there were no differences in overall CHA use by survey version, (pilot) test sample, or clinic location (Fisher’s p’s > .05). Based upon the positive feedback from our subsamples, and the vast similarity of preliminary and final CHAPS versions on all other factors assessed in the survey (i.e., age, sex, race, etc.; p’s ≥ .05), data were pooled for analysis.

### Qualitative data

#### Scale development and assumptions

Despite our high response rates, some items had relatively high rates of missing data, including herbal use (18.3%), BMI (11.6%), alcohol (11%), and income (12.3%). However, there were no consistent visual subgroup patterns of missing data, suggesting missing data were likely missing at random or missing completely at random. Further sensitivity analysis was conducted to estimate the effect of missing data on the relationships of many potential correlates with CHA use for pain; we compared estimates using non-imputed data with multiple imputed data using the Fully Conditional Specification method[92]; estimates were unchanged (not shown), further demonstrating that no factors assessed in the survey were associated with systematic missing data, thus suggesting the appropriateness of MI.

Results indicated very high internal consistency for the SF-GPS[6] (Cronbach’s alpha overall = .93); the internal reliability of each (5-question) subscale was also high (Cronbach’s alpha for the Pain subscale = .89; Feelings subscale = .87; Clinical Outcomes subscale = .83; Activities subscale = .91). There was satisfactory internal reliability for each of the five TTM stages for all (twelve) combined CHA measurements: precontemplation (0.89), contemplation (0.72), preparation (0.75), action (0.70), and maintenance (0.70).

### Descriptive statistics

Sample Characteristics are displayed in Tables 2, 3 and 4. Study participants were predominantly white (92.0%), female (56.9%), and married or cohabitating (57.5%) (Table 2). Participant age ranged from 22 to 88 years, averaging 55.6 (SD = 13.6) years (Table 2). Over 40% of participants were disabled (43%; Table 2) and/or obese (44%, mean BMI = 33.4±31.5; Table 3), and over 55% reported no exercise during the last month (Table 3). Nearly half did not consume alcohol within the past year (46.8%) (Table 3). The majority of our sample (93%) was experiencing (chronic) pain for twelve weeks or more at the time of survey completion, and had five or more health conditions (56%, Mean = 5.4±3.1) (Table 3). Additionally, 44% used opioids and 31% used other prescription medications (Table 3), while 66% used at least one CHA for pain, with 48% using at least one CHA for greater than 6 months (Table 4). Although 34% had not used any CHAs for pain, 21% intended to begin using at least one CHA for pain within 6 months (Table 4).

**Table 2:** Demographics of Appalachian pain patients, Complementary Health Approaches for Pain Survey (CHAPS), WV, 2014–2016.

Characteristic	N	%
Total Sample	301	
Age in years (Mean (SD))	55.6	13.6
18–29	11	4%
30–44	49	17.8%
45–64	147	53.5%
65+	68	24.7%
Gender		
Male	119	43.1%
Female	157	56.9%
Race/Ethnicity		
Non-Hispanic White	253	92%
Other Race	22	8%
Education		
<12^th^ grade	31	11.3%
HS/GED	93	33.9%
Some College/Associate’s/ Technical training	100	36.5%
≥Bachelor’s degree	50	18.3%
Employment		
Employed/Student/ Homemaker	82	29.8%
Retired	58	21.1%
Unemployed	16	5.8%
Disabled	119	43.3%
Military Status*		
Served or serving in military	26	20%
Never served	104	80%
Marital Status		
Married/Cohabitating	158	57.5%
Single	41	14.9%
Divorced/Sep/Widow	76	27.6%
Household Income		
<$25,000	88	29.2%
$25,001–50,000	72	23.9%
$50,001–75,000	39	13%
$75,001+	37	12.3%
Don’t know/Missing	65	21.6%

Note: Column Percentages shown.

*Assessed after initial pilot-test wave (N = 130)

**Table 3:** Lifestyle and Health characteristics of Appalachian pain patients, Complementary Health Approaches for Pain Survey (CHAPS), WV, 2014–2016.

Characteristic	N	%
Alcohol		
None in past year	141	46.8%
<2–3 times per month	87	28.9%
≥1 time per week	40	13.3%
Missing	33	11%
Smoker		
Never	109	39.9%
Current	75	27.5%
Former	89	32.6%
Exercise in past month	119	43.6%
Number of minutes p/wk^a^ (Mean (SD))	196.1	399.1
BMI+ (Mean (SD))	33.8	31.5
≤ 25	53	17.6%
25.1–29.9	81	26.9%
30–34.9	66	21.9%
35+	66	21.9%
Missing	35	11.6%
Chronic Pain		
Currently experiencing	270	93.4%
Experienced in the past	283	97.6%
Global Pain Scale^b^		
Total (Mean (SD))	50.6	20.6
*Pain*	15.5	4.5
*Feelings*	10.7	6.3
*Clinical Outcomes*	13.8	6.3
*Activities*	12.2	7.4
Prescription medication use for pain management*		
None	48	24.9%
Opioids	85	44%
Other Rx	60	31.1%
Number of Health Conditions		
0–1	20	7%
2	27	10%
3	39	14%
4	38	14%
5+	155	56%
Total (Mean (SD) (range)	5.4 (0–16)	3.1
Pain syndromes^c^	2.3 (0–7)	1.58
Mental Health conditions^d^	0.84 (0–2)	0.87
Injury^e^	0.48 (0–2)	0.7
Other conditions^f^	1.4 (0–7)	1.4

Note: Column Percentages shown.

*Assessed after initial pilot-test wave (N = 193); Sample using opioids for pain may or may not also include use of other Rx

a. Only those who exercised in past month without missing exercise data (N = 76)

b. SF-Global Pain Scale score 0–100, with 100 indicative of greatest impact of pain upon life; Subscales Pain, feelings, clinical outcomes, and activities scores each 0–25

c. Includes: Spine/Back/Neck pain, migraines, tension headaches, Rheumatoid Arthritis, Osteoarthritis, Temporomandibular Jaw Disorder, Knee Pain, Fibromyalgia, and/or Gout

d. Includes depression and/or anxiety

e. Includes broken bones and/or musculoskeletal injury/tissue trauma

f. Includes: Hypertension, heart disease, irritable bowel disorder, renal disorder, asthma, chronic bronchitis, diabetes, cancer, stroke, and/or chronic fatigue syndrome

**Table 4:** Complementary Health Approaches (CHAs) used for pain among Appalachian patients seeking conventional pain management by Health behavior stage of change, Complementary Health Approaches for Pain Survey (CHAPS), WV, 2014–2016.

CHA**	No, and will not (Precontemplation)		No, but intend to within 6 months (Contemplation)		No, but intend to within 30 days (Preparation)		Yes, for less than 6 months (Action)		Yes, for more than 6 months (Maintenance)	
	N	%	N	%	N	%	N	%	N	%
Overall Use^a^	229	85.5	56	20.9	31	11.6	87	32.5	128	47.8
Herbs/Botanicals	152	61.8	5	2	5	2	11	4.5	13	5.3
Vitamins/Minerals	98	38.9	7	2.8	4	1.6	43	17.1	85	33.9
**Probiotics**	**112**	**62.2**	**4**	**2.2**	**4**	**2.2**	**11**	**6.2**	**20**	**11.2**
Other Natural Products	150	60	11	4.4	5	2	22	8.8	40	16
Acupuncture	180	79	10	4.4	2	0.88	5	2.2	8	3.5
Massage Therapy	142	62	19	8.3	8	3.5	25	11	17	7.5
Spinal manipulation/ Chiropractic	164	69.2	10	4.2	5	2.1	19	8	22	9.2
Tai chi/Qi Gong	170	71.4	4	1.7	1	0.42	2	0.84	1	0.42
Yoga	184	78.6	10	4.3	6	2.6	3	1.3	7	3
Meditation	165	68.5	8	3.3	8	3.3	12	5	29	12.1
Other relaxation practices	139	58.7	9	3.8	10	4.2	21	9	34	14.5
Movement therapies	145	60.7	4	1.7	1	0.42	5	2.1	10	4.2

Note: Column Percentages shown; those missing or unaware of CHA definition excluded.

**Herbs/Botanicals N = 246; Vitamins/Minerals N = 252; Probiotics N = 180; Other Natural Products N = 250; Acupuncture N = 228; Massage Therapy N = 229; Spinal manipulation/ Chiropractic N = 239; Tai chi/Qi Gong N = 238; Yoga N = 235; Meditation N = 241; Other relaxation practices N = 237; Movement therapies N = 239

a. N = 268; Includes Herbs/Botanicals, Vitamins and/or Minerals, Probiotics, Other natural products, Acupuncture, Massage therapy, Spinal manipulation/Chiropractic, Tai chi/Qi Gong, Yoga, Meditation, Other relaxation practices, and/or Movement therapies

### Challenges to data collection

Our team faced many challenges in data collection. Difficulty in cancellations due to inclement weather, broken clinic equipment, clinic lockdown, and those related to litigation/suspension of physician licensure resulted in low sampling on a number of scheduled days. We coordinated our visits with clinic administrators on days with heavy patient scheduling; however, most scheduled days resulted in many patient no-shows (30–50%). Upon inquiry, administrators informed us this was common, using the rationale that patients were less likely to come for appointments if they were not currently in pain.

Clinic location did not differ among those refusing to participate (n = 42). We always allowed patients to complete their clinic-administered paperwork before approaching them, and patients often expressed concern regarding limited waiting room time. However, the majority of those indicating concern were willing to complete our survey after we told them they could continue completion in patient rooms, if that was their preference. Although we made every effort to avoid sampling from the same patient population, 4/347 patients approached stated they had completed the survey previously.

## Discussion

Although overall CHA use has been examined in a variety of populations[93–101], the CHAPS is the first concise measurement tool designed to evaluate the prevalence and correlates of CHA use specifically for pain, by length of use. The CHAPS is flexible in that it may be used alongside different pain measurement tools by clinicians and researchers, in addition to other tools designed to measure patient expectations of CHAs[102]. Individual patient pain outcomes vary greatly, propelling efforts toward precision medicine[103]; the CHAPS may serve as a time-saving approach to assess patient CHA use for pain. This may be of particular interest for clinicians possessing openness in identifying individual patient response for non-pharmacological approaches as adjuncts to conventional pain management strategies, as the limited evidence base for these approaches is mostly positive but remains inconclusive[79]. Further, clinicians unfamiliar with several types of CHAs may particularly benefit from use of the CHAPS, since information regarding (evidence-based) CHA practices are not consistently integrated into medical school curricula despite their wide use by a variety of patient populations[104, 105]. For example, nearly half of our rural sample were long-term users of CHAs specifically for pain.

Findings of this pilot study suggest that the CHAPS was readily understandable, acceptable, and feasible to implement in chronic pain patients in WV, and that the SF-GPS, a measurement tool designed to capture the multidimensional experience of pain, was reliable for gauging the impact of pain. The GPS, from which the SF-GPS originated, is a valid measurement tool with high internal reliability; interestingly, reliability coefficients for the SF-GPS in the current study were higher than those previously reported in a sample of college students[6], perhaps reflecting in part differences in population characteristics. In addition, we only assessed approaches believed available in Appalachian rural settings. However, future use of the CHAPS may benefit from the addition or substitution of other CHAs available in different settings.

Although selection bias due to non-response is possible, our use of consecutive sampling, coupled with the high survey response rates, render non-response bias less likely. As in many studies using self-report data, there is also a possibility of recall bias. Researchers wishing to implement the CHAPS or similar surveys should explore barriers to survey implementation present at specific clinics, partially by considering attitudes of the clinic staff and working together to facilitate involvement, as well as considering the available physical space.

## Conclusions

The CHAPS is the first measurement tool to assess correlates of CHA use specifically for pain, and was successfully pilot-tested and implemented in a population with pain. The CHAPS included comprehensive information on a range of factors and constructs, yet required only 12 minutes to complete, and may thus be appropriate for use in future population- and clinic-based survey studies assessing CHA use in patients with chronic pain. Robust survey response rates and high face validity further support use of the CHAPS as a measurement tool in rural populations with chronic pain.

## Supporting information

S1 Appendix The CHAPS measurement tool. (DOCX)

S1 Dataset (CSV)

S1 Codebook Accompanying codebook. (XLSX)
