# Supplementary material for: The Complementary Health Approaches for Pain Survey (CHAPS): Validity testing and characteristics of a rural population with pain
Source: PLoS One. 2018 May 2;13(5):e0196390. doi: 10.1371/journal.pone.0196390 (PMC5931640; doi:10.1371/journal.pone.0196390)
Supplement: S1 Appendix — (DOCX) [file pone.0196390.s001.docx]

**Appendix A. Complementary Health Approaches for Pain Survey**

Chronic pain is often defined as any pain lasting more than 12 weeks. Chronic pain may arise from an initial injury, such as a back sprain, or there may be an ongoing cause, such as illness. Have you **ever** experienced pain lasting 12 weeks or longer?

Yes

No

Are you **currently** experiencing chronic pain as defined in the previous question (pain lasting 12 weeks or longer)?

Yes

No

I have experienced chronic pain before but am not experiencing chronic pain presently

**PLEASE CHECK THE BOX(ES) NEXT TO CONDITIONS YOU HAVE BEEN DIAGNOSED WITH:**

| Hypertension | Chronic Bronchitis | Chronic Back Pain |
| --- | --- | --- |
| Heart Disease | Diabetes | Knee Pain |
| Inflammatory Bowel Disease | Cancer  (Currently being treated) | Fibromyalgia |
| Impaired Renal Function/Kidney Disease | Stroke | Gout |
| Asthma | Rheumatoid Arthritis | Migraines |
| Depression | Osteoarthritis | Tension Headaches |
| Anxiety | Chronic Fatigue Syndrome | Injury: Broken Bone(s) |
| Spine and/or Neck Issues | Temporomandibular Jaw Disorder (TMJ) | Injury: Musculoskeletal or Soft Tissue Trauma (such as whiplash, torn ligament, etc.) |

Please list any other health conditions you have.

________________________________________________________________________________________________________________________________________________________________________________________________________________________________________________________________________________________________________________________________________

**Please check the box in each row to describe your usage of Complementary Health Approaches**

**FOR PAIN:**

| **Treatment/**  **Prevention used**  **for pain?** | **I do not know what this is** | **No, and I do not intend to within the next 6 months** | **No, but I intend to within the next 6 months** | **No, but I intend to within the next 30 days** | **Yes, and I have for less than 6 months** | **Yes, and I have for more than 6 months** | **If Yes, how effective is it in managing your pain?** |
| --- | --- | --- | --- | --- | --- | --- | --- |
| Herbs/Botanicals (such as Echinacea, Black Cohosh, etc.) |  |  |  |  |  |  | ____________________ |
| Vitamins and/or Minerals (such as Vitamin C, Magnesium, etc.) |  |  |  |  |  |  | ______________________________ |
| Probiotics |  |  |  |  |  |  | ____________________ |
| Other natural products (such as fish oil, lecithin, etc.) |  |  |  |  |  |  | ____________________ |
| Acupuncture |  |  |  |  |  |  | ____________________ |
| Massage therapy |  |  |  |  |  |  | ____________________ |
| Spinal manipulation/  Chiropractic |  |  |  |  |  |  | ____________________ |
| Tai chi/Qi gong |  |  |  |  |  |  | ____________________ |
| Yoga |  |  |  |  |  |  | ____________________ |
| Meditation |  |  |  |  |  |  | ____________________ |
| Other relaxation practices |  |  |  |  |  |  | ______________________________ |
| Movement therapies (such as Alexander technique, Rolfing, etc.) |  |  |  |  |  |  | ______________________________ |

Please list any other Complementary Health Approaches you have used **for pain**, if any. Some examples include Ayurveda, healing touch/Reiki, and homeopathy.

____________________________________________________________________________________________________________________________________________________________________________________________________________________________________________________________________________

Please **list any specific herbs/botanicals** that you have used or intend to use **for the treatment or prevention of pain.** Some common examples include peppermint, black cohosh, lavender, kava kava, and ginger.

**____________________________________________________________________________________________________________________________________________________________________________________________________________________________________________________________________________________________________**

Please list any prescriptions you have used **for pain**, if any. If you use a pump or stimulator, please specify which one.

­­­­­­­­­­­­­­­­­­­­­­­­­­­­­­

______________________________________________________________________________________________________________________________________

**PLEASE CHECK THE BOX WHICH BEST APPLIES TO YOU:**

What is your gender?

Male

Female

Other

What is your marital status?

Single

Married

Divorced

Separated

Widowed

A member of an unmarried couple, living together

What is your race/ethnicity?

White/Caucasian

Hispanic/Latino

African American

Native American or American Indian

Asian/Pacific Islander

Two or more races

Other_________________________________

What is the highest degree or level of school you have completed?

No schooling

Nursery school to 8^th^ grade

Some high school, no diploma

High school diploma/GED

Some college credit, no degree

Associate degree or Trade/Technical/Vocational training

Bachelor’s degree

Master’s degree

Doctorate or equivalent professional degree

What is your work status?

Employed for wages

Self-employed

Student

Disabled

Not Working/Unemployed

Retired

A Homemaker

What is your military status?

Veteran

Active duty

Guard/Reserves

Served without Veteran Status (kicked out/dishonorably discharged)

Never served in the Armed Forces

What is your annual household income, from all sources?

Don’t know

Less than $25,000

$25,001 - $50,000

$50,001 - $75,000

$75,001 or more

What is your cigarette smoking status?

Former Smoker

Current Smoker

I have never smoked

During the past month, did you participate in any physical activities or exercises such as yoga, aerobics, or walking for exercise?

No (Go to next page.)

Yes

If yes, what type (for example, walking)?

____________________

____________________

____________________

If yes, how many days per week did you exercise? ____________________________________________________________

If yes, how many minutes did you exercise per day?

____________________________________________________________

During the last 12 months, how often did you usually have any kind of drink containing alcohol? **By a drink we mean half an ounce of absolute alcohol (e.g. a 12 ounce can or glass of beer or cooler, a 5 ounce glass of wine, or a drink containing 1 shot of liquor)**. Choose only one.

Every day

5-6 times a week

3-4 times a week

Twice a week

Once a week

2 to 3 times a month

3 to 11 times in the past year

1 or 2 times in the past year

None

**PLEASE FILL IN THE BLANK:**

What is your age? What is your weight?

­­­­­­__________years __________lbs.

What is your current height?

If you are a person with a physical disability (of your legs), please state your lifetime maximum height.

­­­­­­__________ feet __________ inches

*Please place this form in the drop box located in the waiting room. Thank you for taking our survey!*
